# Prevalence of congenital anomalies and related factors in live births in Zahedan, Southeast of Iran: A cross-sectional study

**DOI:** 10.18502/ijrm.v21i8.14020

**Published:** 2023-09-20

**Authors:** Azam Asemi-Rad, Zahra Heidari, Hamidreza Mahmoudzadeh-Sagheb, Yousef Mehdipour, Bita Moudi, Nadia Sheibak, Saeid Ebrahimi

**Affiliations:** ^1^Department of Anatomical Sciences, School of Medicine, Zahedan University of Medical Sciences, Zahedan, Iran.; ^2^Cellular and Molecular Research Center, Zahedan University of Medical Sciences, Zahedan, Iran.; ^3^Department of Histology, School of Medicine, Zahedan University of Medical Sciences, Zahedan, Iran.; ^4^Infectious Diseases and Tropical Medicine Research Center, Resistant Tuberculosis Institute, Zahedan University of Medical Sciences, Zahedan, Iran.; ^5^Department of Health Information Technology, School of Paramedical Sciences, Torbat Heydarieh University of Medical Sciences, Torbat Heydarieh, Iran.; ^6^Department of Health Information Technology, Zahedan University of Medical Sciences, Zahedan, Iran.

**Keywords:** Congenital anomalies, Hospitalization, Iran, Live birth, Prevalence, Risk factors.

## Abstract

**Background:**

The term congenital anomalies (CAs) refers to structural or functional abnormalities at the time of conception. Approximately 12 deaths related to congenital disabilities occur in every 10,000 babies born.

**Objective:**

This study aimed to evaluate the prevalence and associated factors of single and multiple CAs in live births in Zahedan, Southeast Iran.

**Materials and Methods:**

This cross-sectional study was conducted on 59,087 live births in a referral hospital in Zahedan located in the southeast of Iran from 2009 to 2019. All live births were examined by pediatricians and the CAs and categorized based on the international classification of diseases.

**Results:**

Of 59,085 live births, at least 883 had a significant anomaly, and the prevalence rate of CAs was about 149 per 10,000. Anomalies of the nervous (24.1%) and cardiovascular systems (21.10%) were the most frequent, occurring in 213 and 187 of the live births, respectively. Spina bifida is the most common anomaly of the central nervous system. The most common anomalies in the cardiovascular system were unspecified heart malformations (17.1%), cardiovascular malformations (18.7%), and patent ductus arteriosus (11.7%). Significant correlations were found between the parent's consanguinity marriage, the mother's age, an existing anomaly in the family, and relatives in single and multiple CAs (p = 0.02, p = 0.02, p < 0.001, p = 0.01, respectively).

**Conclusion:**

The prevalence of CAs was 149 per 10,000 live births. The highest prevalence of CAs was related to the central nervous system. Increasing the public's knowledge about fetal defects can reduce the prevalence of CAs.

## 1. Introduction

Congenital anomalies (CAs) represent a group of abnormalities that occur during pregnancy and are associated with prenatal death, childhood mortality, and adolescent disability (1, 2). Since 1960, general supervision for the emergence of infants with CAs has been carried out in various populations worldwide. It has been shown that the prevalence of preterm labor and CA noticeably varies in different countries (3, 4). In the United States, the prevalence rate of CA in newborns is 3% (3). According to the European Congenital Abnormalities Organization, the average rate of birth defects was 24.26 per 1000 births from 2010-2014 (5). The prevalence of CA in Iran has been reported in some studies. In northern Iran, the prevalence rate of congenital heart abnormalities was 6-8 per 1000 live births (6). In another study in the Northeast, Iran, the CA rate was about 29.11 per 1000 live births (7).

Typically, the most common causes of neonatal mortality are disorders associated with developmental defects (1, 2). As defined by the World Health Organization (WHO), these defects include single or multiple structural defects, such as cleft lip, and cleft palate, or functional defects that contain biochemical and molecular defects that can be identified at birth (8). CA is divided into 2 categories based on severity, major and minor defects. Major defects are defined as anatomical defects that affect a person's life and appearance. Minor defects are structural changes that do not require treatment or are recovered with simple methods (9). Based on the categories provided by the WHO, CA includes defects in the nervous system, eyes, ears, face and neck, cardiovascular system, respiratory system, cleft lip and palate, gastrointestinal system, urinary and genital system, musculoskeletal system, clinical syndromes, and chromosomal abnormalities. Each category contains a sub-division (10).

The prevention of CA requires background knowledge. The prevalence rate of these deficiencies can contribute somewhat to their prevention (8). The majority of CAs have unknown reasons (11) and are caused by different factors, like physical and chemical environmental factors as well as various maternal factors, such as age, type of pregnancy, delivery type, and maternal health (12).

According to the fact that the estimation of the prevalence rate of CA is crucial to developing prevention strategies, and to the best of our knowledge, there have been few or no studies directly related to the prevalence of CA and predisposing risk factors in Southeast Iran. This study aimed to evaluate the prevalence of CAs and related risk factors in live births from 2009-2019 in Zahedan, Southeast Iran.

## 2. Materials and Methods

This cross-sectional hospital-based study was conducted retrospectively, assessing the medical records of all newborns delivered in the Ali-Ibn-Abitaleb referral hospital of Zahedan, Iran over a 10 yr period, from April 2009-2019. Inclusion and exclusion criteria were as follows, cases with stillbirth were eliminated from the study; pediatricians examined all live births, and anomalies were registered. Newborns with at least one diagnosed CA were enrolled in the study. For this study, CA was defined as major structural defects that existed at birth or in infancy, either clinically or through screening methods. These anomalies were classified using the International Classification of Disease Code, version 11 (Table I) (10).

The information obtained consisted of sex, birth age, birth weight, mother's age, delivery type, pregnancy type, mother's underlying disease, mother's medicine consumption, parents smoking and drug usage, consanguinity marriage between parents, the existence of CA in family and relatives, and history of hospitalization.

**Table 1:** Types of CAS observed in live births from 2009-2019


**Type of CA**	**Q code**	**Total CA**	**Per 10,000 live births**
**Nervous system**	Q00-07	213 (24.12)	36.40
**Cardiovascular system**	Q20-28	187 (21.17)	31.64
**Eyes, ears, face, neck**	Q10-18	177 (20.04)	29.95
**Digestive system**	Q38-45	125 (14.15)	21.15
**Cleft lip and palate**	Q35-37	53 (6.00)	8.97
**Urinary system**	Q60-64	38 (4.30)	6.43
**Musculoskeletal **	Q65-79	34 (3.85)	5.75
**Genital organ**	Q50-56	31 (3.51)	5.24
**Respiratory system**	Q30-34	16 (1.81)	2.70
**Chromosomal abnormalities**	Q90-99	6 (0.67)	1.01
**Other CAs**	Q80-89	3 (0.33)	0.50
CA: Congenital anomalies. Q codes: CAs are classified using the International Classification of Disease code, version 11			

### Ethical considerations

The Ethics Committee of Zahedan University of Medical Science, Zahedan, Iran approved this study (Code: IR. ZAUMS.REC.1398.076). A written consent form was signed by each participants.

### Statistical analysis 

A student *t* test was used to determine whether 2 independent groups had significantly different means. Quantitative variables were compared using the ANOVA test, while qualitative variables were compared using the Chi-square test. Finally, the results are reported as mean ± SEM. The statistical tests were conducted using SPSS software version 20. Statistical significance was defined as p ≤ 0.05.

## 3. Results

In the current study, 705 newborns with a single anomaly and 178 newborns with multiple anomalies were enrolled; 523 cases were male and 358 were female, while the gender of the 2 cases were unknown due to ambiguous genitalia.

**Figure 1:**
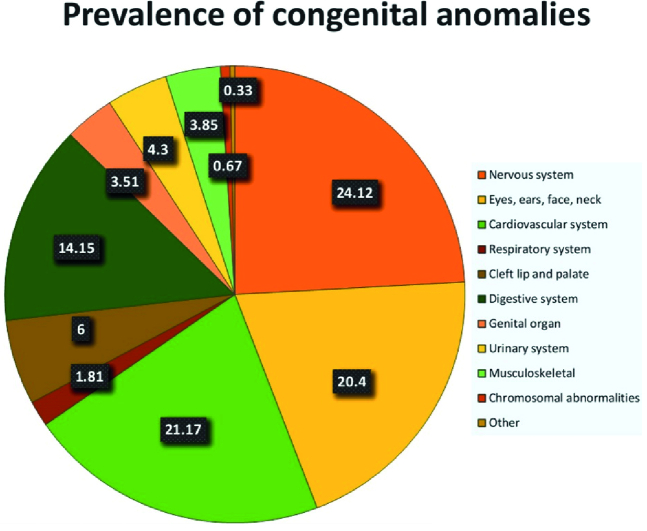
The prevalence of CAs in live births during 2009-2019, Zahedan, Iran. The data in the figure shows the percentage of anomalies in each group. CAs: Congenital anomalies.

The results of this study indicated that the obvious CAs were 883 in 59,085 neonate live births in April 2009-2019, giving an overall prevalence rate of 1.49% (Table I). As reported in table II, CA in the nervous system was a frequent anomaly and after the other CAs and chromosomal abnormalities, malformations of the respiratory system had the lowest prevalence. CAs in the cardiovascular system and the eyes, ears, face, and neck was the second and third highest after the nervous system, respectively. Moreover, the CAs of the digestive system is more frequent than the malformation of the cleft lip and palate (Table I, Figure 1).

A large percentage of CAs in the nervous system are affected by unspecified spina bifida. The most common anomalies in the cardiovascular system were unspecified malformations of the heart, unspecified malformations of the cardiovascular system, and patent ductus arteriosus. Moreover, unilateral cleft lip showed a higher prevalence compared to bilateral disorder.

Out of all anomalies in the eyes, ears, face, and neck, malformations of the ear causing hearing impairment was the most common. Further, the most prevalent anomaly in the genital system was hypospadias. In this study, congenital hydronephrosis represented the highest percentage of urinary system anomalies and the congenital diaphragmatic hernia also had the highest number of musculoskeletal anomalies. We found that the anomaly of pleura folds was the most common among the abnormalities affecting the respiratory system. Eventually, Down syndrome became the most prevalent anomaly in the category of other CAs (Table II).

More detailed demographic data of newborns enrolled in the present study, including newborns' sex, birth age, and weight, delivery, and pregnancy types, and information about their parents, such as maternal age, consanguineous marriage, maternal health, and family history, are presented in table III.

Statistical analysis showed no correlation between CAs and sex, birth age, pregnancy type, or delivery type (p = 0.40, Table III). No significant relationship was observed between the type of CA and medicine consumption (p = 0.09). Besides underlying diseases, smoking, and drug usage do not correlate with the type of CA (respectively, p = 0.20, p = 0.50). In contrast, a significant correlation was observed between CA and the parent's consanguinity marriage (p = 0.02). The probability of having a baby with multiple CAs was significantly higher in mothers over 35 (p = 0.02).

A significant relationship existed between an existing anomaly in the family and relatives with CA (p < 0.001, p = 0.01). Furthermore, hospitalization significantly correlates with multiple anomalies (p < 0.001; Table III).

**Table 2:** Types and numbers of CAs observed in live births from 2009-2019


**CAs**	**Number**	**Types of anomalies**	**Incidence**
	Spina bifida, unspecified	41 (19.25)
	Microcephaly	38 (17.84)
	Malformations of aqueduct of Silvius	36 (16.90)
	Lumbar spina bifida without hydrocephalus	43 (20.19)
	Cervical spina bifida with hydrocephalus	31 (14.55)
**Nervous system**	213	Congenital hydrocephalus	24 (11.27)
			Congenital malformations of the ear cause impairment of hearing	31 (17.51)
			Congenital malformation of face and neck, unspecified	20 (11.30)
			Congenital malformation of the eye, unspecified	16 (9.04)
			Congenital glaucoma	7 (3.95)
			Microstomia	10 (5.65)
	Other branchial cleft malformations	8 (4.52)
	Congenital malformation of ear ossicles	13 (7.35)
	Congenital malformation of the inner ear	10 (5.65)
	Microtia	16 (9.04)
	Congenital malformation of ear, unspecified	22 (12.43)
	Congenital lens malformation, unspecified	10 (5.65)
**Eyes, ears, face, neck**	177	Congenital malformation of face and neck, unspecified	14 (7.91)
			Ventricular septal defect	19 (10.16)
			Tetralogy of Fallot	10 (5.35)
			Stenosis of the pulmonary artery	6 (3.21)
			Stenosis of aorta	5 (2.67)
			Situs inversus	4 (2.14)
			Patent ductus arteriosus	22 (11.77)
			Ebstein anomaly	5 (2.67)
			Congenital mitral insufficiency	7 (3.74)
			Discordant ventriculoarterial connection	5 (2.67)
			Dextrocardia	9 (4.81)
			Atrioventricular septal defect	4 (2.14)
	Atrial septal defect	16 (8.56)
	Other congenital malformations of the pulmonary artery	3 (1.61)
	Other congenital malformations of the tricuspid valve	5 (2.67)
	Unspecified congenital malformations of the system	35 (18.72)
**Cardiovascular system**	187	Unspecified congenital malformations of the heart	32 (17.11)
			Fissured, notched, and cleft nose	2 (12.50)
			Anomaly of pleura folds	5 (31.25)
			The accessory lobe of the lung	3 (18.75)
			Agenesis and underdevelopment of nose	3 (18.75)
		**Respiratory system**	16	Congenital malformation of the nose	3 (18.75)
					Cleft lip and palate	5 (9.43)
					Cleft palate, unspecified	18 (33.97)
					Unspecified cleft palate with unilateral cleft lip	5 (9.43)
					Unspecified cleft palate with bilateral cleft lip	6 (11.32)
			Cleft lip, unilateral	10 (18.87)
		**Cleft lip and palate**	53	Cleft lip, bilateral	9 (16.98)
					Hirschsprung disease	21 (16.80)
					Other congenital malformations of the gallbladder	4 (3.20)
					Meckel diverticulum	3 (2.40)
	Atresia of the esophagus without fistula			15 (12.00)
	Atresia of bile ducts			6 (4.80)
	Cystic disease of the liver	2 (1.60)
	Congenital fistula of rectum and anus	8 (6.40)
	Congenital hypertrophic pyloric stenosis	18 (14.40)
	Congenital malformations of intestinal fixation	3 (2.40)
	Congenital absence, atresia, and stenosis of the small intestine	12 (9.60)
	Congenital absence, atresia, and stenosis of jejunum	13 (10.40)
**Digestive system**	125	Congenital absence, atresia, and stenosis of the anus	20 (16.00)
			An undescended testicle, bilateral	5 (16.13)
			Other congenital malformations of the fallopian tube a	7 (22.58)
			Indeterminate sex, unspecified	5 (16.13)
			Hypospadias	10 (32.26)
		**Genital organ**	31	Embryonic cyst of the fallopian tube	4 (12.90)
					Congenital vesico-uretero-renal reflux	2 (5.27)
					Polycystic kidney, autosomal recessive	3 (7.89)
					Polycystic kidney, autosomal dominant	3 (7.89)
					Hyperplastic and giant kidney	6 (15.79)
			Congenital hydronephrosis	14 (36.84)
			Other specified congenital malformations of the kidney	5 (13.16)
	Other congenital malformations of the ureter	3 (7.89)
**Urinary system**	38	Congenital vesico-uretero-renal reflux	2 (5.27)
			Polydactyly, unspecified	7 (20.59)
			Other congenital malformations of the lower limb(s)	4 (11.77)
			Epidermolysis bullosa, unspecified	2 (5.88)
			Congenital ichthyosis, unspecified	2 (5.88)
			Craniosynostosis	3 (8.82)
			Congenital diaphragmatic hernia	13 (38.24)
		**Musculoskeletal**	34	Congenital complete absence of lower limb(s)	3 (8.82)
**Chromosomal abnormalities**	6			Down syndrome, unspecified	6 (100)
**Other CAs**	3			Other specified congenital malformations of the integumentary system	3 (100)
Data presented as n (%). CAs: Congenital anomalies			

**Table 3:** Factors associated with CAs from 2009-2019


**Variables**		**Single anomaly**	**Multiple anomalies**	**P-value**
**Sex**				
	**Male**	413 (46.77)	110 (12.46)	
	**Female**	290 (32.84)	68 (7.70)	
	**Ambiguous genitalia**	2 (0.23)	0 (0)	0.50
**Birth age (wk)**				
	**Pre-term**	142 (16.08)	49 (5.55)		
	**Term**	520 (58.89)	124 (14.04)	
	**Post-term**	43 (4.87)	5 (0.57)	0.10
**Birth weight (gr)**				
	**< 2500**	149 (16.87)	64 (7.24)		
	**≥ 2500**	556 (62.96)	114 (12.91)	0.30
**Mother's age (yr)**				
	**≤ 35**	659 (74.63)	166 (18.80)	
	**> 35**	46 (5.21)	12 (1.36)	0.02*
**Delivery type**				
	**Natural**	652 (73.84)	162 (18.35)	
	**Cesarean**	53 (6.00)	16 (1.81)	0.40
**Pregnancy type**				
	**Natural**	683 (77.35)	173 (19.59)	
	**Assisted**	22 (2.49)	5 (0.57)	0.60
**Consanguineous marriage**				
	**Yes**	404 (45.75)	125 (14.16)	
	**No**	301 (34.09)	53 (6.00)	0.02*
**Mother's underlying disease**				
	**Yes**	86 (9.74)	33 (3.74)	
	**No**	619 (70.10)	145 (16.42)	0.40
**Medicine consumption**				
	**Yes**	70 (7.93)	34 (3.85)	
	**No**	635 (71.91)	144 (16.31)	0.09
**Smoking**				
	**Yes**	29 (3.28)	0 (0)	
	**No**	676 (76.56)	178 (20.16)	0.20
**Drug use**				
	**Yes**	28 (3.17)	9 (1.02)	
	**No**	677 (76.67)	169 (19.14)	0.50
**History of an anomaly in the family**				
	**Yes**	273 (30.92)	66 (7.47)	
	**No**	432 (48.93)	112 (12.68)	0.005*
**History of an anomaly in relatives**				
	**Yes**	295 (33.41)	95 (10.76)	
	**No**	410 (46.43)	83 (9.40)	0.01*
**Hospitalization**				
	**Yes**	82 (9.30)	50 (5.66)	
	**No**	623 (70.55)	128 (14.49)	0.001*
Data presented as n (%). Student* t* test, *Significant difference between groups (p ≤ 0.05). CAs: Congenital anomalies				

## 4. Discussion

The current study showed that the total prevalence of CA in live birth was 149 per 10,000 births (1.49%) in Zahedan. Anomalies of the nervous and cardiovascular systems were the most frequent anomalies in live births and spina bifida was the most common anomaly of the central nervous system. The most common anomalies of the cardiovascular system were unspecified heart malformations, cardiovascular malformations, and patent ductus arteriosus. We found significant correlations between the parent's consanguinity marriages, the mother's age, an existing anomaly in the family, and relatives with the presence of single and multiple CAs.

By reviewing the hospital centers, the WHO has reported a rate of 2.20% for congenital malformations (13). This incidence rate is about 2-5% in Europe and the United States (14). According to a report on the prevalence of live birth with CAs in Europe, the prevalence was more than 10.4 per 1000 live births (15). A study of the prevalence of CAs in newborns in São Paulo indicated a prevalence of 1.6 per 100 live births (16). A lower rate in the current study may be due to lower health care service usage for delivery, considering some indigenous beliefs.

Also, midwives may not have registered anomalies at the delivery facility centers. The current study was a partial estimation about the physical examination of newborns, and additional disabilities diagnosed with age or abnormalities that cause death during the fetal period were not counted. Besides, some abnormalities present at birth are not obvious until a while later. In this study, the frequency of cases with CAs in male neonates was higher than in female neonates. 2 other studies also reported a higher prevalence of CA in male newborns (9, 17).

According to the previous results, nervous system anomalies are the most frequent defects. The study's findings showed that the incidence of CA in the central nervous system was about 3.64 per 1000 births. The prevalence of nervous system defects reported about 0.4 per 1000 births (15). In a study in Iran, it was demonstrated that the prevalence of neural tube defects was about 1.01-8.29 per 1000 live births (4).

The most prevalent system affected by CA after the nervous system is the cardiovascular system, the eyes, ears, face, neck, and digestive system, respectively, with a prevalence rate between 21 and 31 per 10,000 live births.

In a study that analyzed the prevalence of congenital heart disease (CHD)-related articles worldwide, the result demonstrated that the prevalence of CHD was 9.4 in 1000 live births and globally increased and changed around the world with a considerable increase in Asia (18). The age of CHD diagnosis and the method used for screening are the main reasons for the difference in CHD prevalence (19). It is indicated that the possibility of a CHD diagnosis increases as the gestational age increases (20).

Around the world, the cleft palate with or without a cleft lip occurs in about one in every 700 live births (13). The global prevalence of cleft palate in every 1000 live births was estimated about 0.33 and the prevalence of cleft lip and palate was 0.45 per 10,000 live births (21), and our results demonstrated 8.97 per 10,000 live birth.

The current study showed that the prevalence of CA in the urinary system, musculoskeletal system, and genital system affects about 6.43 to 5.24 per 10,000 live births. The prevalence of congenital disorders of the urinary system among newborns in the US was estimated at 2% (22). In another study in Egypt, the CA of the genital system was about 5.4% (23). Abnormalities in the kidney and urinary systems are associated with lower gestational age and genetic disorders, so they are diagnosed earlier in pregnancy (22).

The current study found that the respiratory system, chromosomal abnormalities, and other CAs had a lower prevalence rate. Notably, this information is acquired from the hospital's information technology system and includes only a fraction of the newborn population.

The difference between the results of the abovementioned studies may be due to low socioeconomic status and education. Although increasing public information accompanied by access to healthcare may lead to increased detection of minor abnormalities, termination of pregnancy with severe anomalies can also decrease incidence (18).

No significant relationship was observed between CA and sex, birth weight, type of delivery, smoking, or drug usage. Consanguinity marriage between parents, a person with CA in the family, and relatives had a significant relationship with multiple anomalies, and hospitalization was more likely in cases with multiple anomalies. Consanguinity marriage is known as a risk factor for CA. It is shown that the risk of giving birth to a baby with CA in parents with a consanguinity marriage is higher than in others (24, 25). The prevalence of consanguineous marriage in different parts of Iran is around 37.40% (26), and the difference in the probability of risk obtained in different studies may be due to differences in the prevalence of consanguineous marriage in different regions.

Moreover, consanguinity marriage is common in Iran, especially among the ethnicities, including Sistani and Baluch, which have a relatively high frequency. Previous studies have indicated that the consanguinity of parents could increase the risk of CAs (27). In this study, the frequency of CAs was higher in infants born to women aged over 37 yr, and this difference was statistically significant. The risk of fetal chromosomal abnormalities increased with maternal age (28).

The current study demonstrates the prevalence rate of CAs in live birth in Zahedan, Iran, where there is no report about the CA. The study strength was an adequate time period of around 10 yr, which allowed the study of abnormalities in 59,085 live births. However, we may have significantly underestimated the actual incidence of these anomalies in the general population. Moreover, we did not include abortions and stillbirths in this study. However, the prevalence of CA is higher among aborted fetuses and stillbirths (29). Also, the anomaly data may not be registered at the delivery facility centers by midwives.

## 5. Conclusion

Congenital disabilities are the leading cause of death in children under the age of 5. Therefore, to prevent and evaluate birth defects accurately, obtaining data about the prevalence of birth defects is necessary. Using the obtained data, effective interventions can be performed on some defects, such as NTDs, which can be prevented by taking folic acid during pregnancy (29).

The current study estimated the prevalence of CAs at 14.6 per 1000 live births. Because some families do not utilize medical services for delivery, as well as the initial assessments of the newborn, it is necessary to have a preconception visit. Also, increasing general knowledge about the leading causes of fetal defects and prenatal screening can progressively reduce the apparent prevalence of CAs. Furthermore, after alerting physicians concerning the importance of visiting newborns and diagnosing CAs, training the nurses and midwives is essential for accurately recording cases and having the exact statistics, because this region is a high-traffic border between the countries. Also, by creating a monitoring system, the precise pattern, the possible etiology, and the prevalence of abnormalities in the general population could be determined. It must be considered in childbearing and population-youth policies.

##  Conflicts of Interest

The authors declare that there is no conflict of interest.
